# Applications of cell sorting in biotechnology

**DOI:** 10.1186/1475-2859-5-12

**Published:** 2006-03-21

**Authors:** Diethard Mattanovich, Nicole Borth

**Affiliations:** 1 University of Natural Resources and Applied Life Sciences Vienna, Department of Biotechnology, Institute of Applied Microbiology, Muthgasse 18, A-1190 Vienna, Austria; 2 School of Bioengineering, University of Applied Sciences FH-Campus Vienna, Muthgasse 18, A-1190 Vienna, Austria

## Abstract

Due to its unique capability to analyze a large number of single cells for several parameters simultaneously, flow cytometry has changed our understanding of the behavior of cells in culture and of the population dynamics even of clonal populations. The potential of this method for biotechnological research, which is based on populations of living cells, was soon appreciated. Sorting applications, however, are still less frequent than one would expect with regard to their potential. This review highlights important contributions where flow cytometric cell sorting was used for physiological research, protein engineering, cell engineering, specifically emphasizing selection of overproducing cell lines. Finally conclusions are drawn concerning the impact of cell sorting on inverse metabolic engineering and systems biology.

## Introduction

The establishment of flow cytometry as the first single cell analysis method with the potential to describe the distribution of cellular properties within a large number of cells has considerably changed our knowledge of cell populations. Before the establishment of flow cytometry our understanding of the immune system was limited to the knowledge that there are leukocytes, monocytes and macrophages, but the complex interplay of the different B- and T-cells, effector and killer cells was only resolved when monoclonal antibodies became available and were used for immunophenotyping by flow cytometry. Today the most frequent use of flow cytometry is still in medical diagnosis. Prior to flow cytometry it was also common belief that in a single strain culture all cells behave homogeneously. Flow cytometry has shown that in reality quite surprising variations are present, which may be caused both by genetic or epigenetic alterations or by differences in the state of individual cells which will diversify their reaction to present culture conditions. Analysis of cells in culture has shown that with the exception of DNA content, all other cellular components are distributed over a wide range, which is the reason why such parameters are usually presented on a logarithmic scale in flow cytometry histograms. This variation of cellular properties is of special interest for strain improvement purposes, as it allows the sorting of cells with diverging and potentially optimized properties.

**Figure 1:**
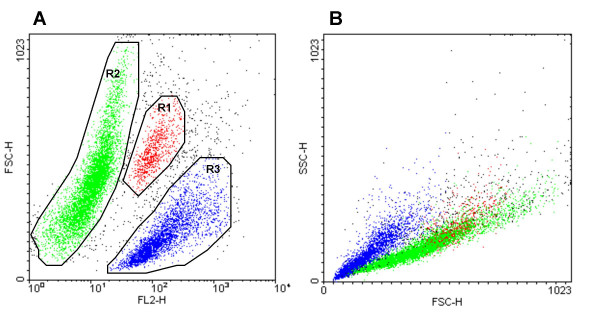
**Multiparameter analysis methods**. By gating on different subpopulations, their properties with regard to additional parameters can be determined. Most commercial flow cytometers can measure between 4 and 8 fluorescence signals in addition to forward scatter (FSC) and side scatter (SSC). As an illustration of the compexity of multiparameter data, the graph below shows a sample of yeast cells stained for viability with ethidium bromide. A: ethidium bromide fluorescence (FL2) against cell size (FSC). B: size (FSC) against granularity characteristics (SSC). Cells marked in panel A as belonging to distinct subpopulations are identified by the same colors in panel B.

The principle of flow cytometry can be described as a fluorescent microscope without morphological resolution where the cells travel in a liquid stream instead of resting on a slide. Each single cell, as it passes the exciting light and the measuring optics, sends out a number of signals, including the size and structure related forward and side scatter and the fluorescent signals, which in turn are dependent on the staining procedure that has been used. These signals are measured and stored for each individual cell. One of the revolutionary properties of flow cytometry is the possibility to measure correlated data: by staining with several fluorescent labels, it is possible to obtain the distribution of each of these parameters within the population, but also their interrelationship (Fig. 1). This information can of course also be obtained using a fluorescent microscope and image analysis, but only from a limited number of cells. With flow cytometry, analyzing 10^4 ^cells is standard procedure and it is possible to look at millions of cells without much trouble. This feature is especially important for biotechnological applications, because it is usually the rare cell which is of interest: the cell with altered properties, a higher production rate, better metabolic parameters or containing the protein with a higher binding affinity. Such rare cells with altered properties are the main target of cell sorting. To be able to sort, it is a prerequisite that flow cytometric methods have been established that allow the characterization of a specific cellular property. Analysis by flow cytometry will provide the distribution of parameters within the population and thus tell the researcher how good the chances are to find a cell with outstanding properties. Development of a suitable flow cytometry protocol also paves the way to the establishment of a good sorting protocol. Indeed, analytical procedures have quickly found their way into biotechnological research. Frequently analyzed cellular properties include: viability and cell performance in recombinant fermentations, cell growth under different culture conditions, characterization of heterogeneous populations from the environment or waste treatment plants, characterization of production cell lines with respect to product content and other cellular properties, to name only a few. The main goal of these analyses is to describe and understand cellular behavior, in the hope that a better understanding will allow to select cells with a desired property. Surprisingly, although the potential of flow cytometry in cell line characterization is widely accepted, the use of cell sorting as a tool to optimize cell lines and protein properties is still lagging behind, with only few publications that take advantage of its obvious potential.

In principle, the term cell sorting can be used for any technique that separates cells according to their properties. Such techniques include panning [1], fluorescence activated cell sorting (FACS) [2] or magnetic cell sorting (MACS) [3]. Novel developments based on dielectrophoresis to move cells in microfluidic devices should be mentioned [4,5] but their application in biotechnology still needs to be shown. Both panning and magnetic cell sorting can be used for the selection of cells according to the expression of a surface molecule, because only such surface expressed molecules can be detected by these techniques using specific ligands. Both techniques also are bulk methods, that allow the rapid isolation of a large number of cells, without the ability to fine-grade according to expression level. Basically, they operate with a yes or no decision, but are unable to differentiate e.g. between low and high producing cells [6]. The only method that allows both single cell selection as well as a (relative) quantification of the production of a specific compound, is flow cytometry. In addition, intracellular markers such as green fluorescent protein (GFP) or markers that describe a physiological activity, such as an enzyme activity or membrane potential, can only be measured and used for sorting by flow cytometry. Even though not a bulk method, desirable cells can be isolated quickly and efficiently. With a traditional cell sorter one can easily look at a few million cells to find the rare outstanding performers (17 minutes at 1000 cells per second). With the modern high speed sorters this will take less than a minute.

In the following, utilization of cell sorting in biotechnology will therefore be highlighted with the main emphasis on fluorescence activated cell sorting. The actual selection of single cells is achieved by different types of sorters, the most frequently used ones being jet-in-air sorters. The liquid stream with the cells, after passing the laser light and the optics, is split up into defined droplets. The droplets containing a cell to be sorted is charged and then deflected into either a separate tube or directly into the individual wells of a microtiter plate. Table 1 provides an overview of typical applications of cell sorting in biotechnology.

**Table 1:** Overview of cell sorting applications in biotechnology

General aim	Sorting target	Selected examples	References
Physiological research	Viability, vitality	bacteria, yeasts	[2-8]
Protein engineering	ligand binding	antibody surface display	[22-24]
		peptide surface display	[15, 16, 26-29]
	enzyme engineering	intra- and extracellular enzymes	[30-33]
Cellular properties	cell hybridization, cloning	yeast hybridization, library cloning	[37, 38]
	promoter trapping	bacteria	[45, 46]
	robustness	acid tolerance	[51]
	process related properties	high cell density, low growth rate	[51-55]
Overproduction	product stained by immunofluorescence	protein	[1, 56-69]
	Autofluorescence of product	alkaloids	[76]
	Unspecific staining	FITC/antibiotic production	[78]

## Physiological research

Flow cytometry, but also cell sorting have become valuable tools for physiological research in biotechnology. Cell sorting allows a more in depth characterization of cells with specific properties observed in flow cytometric analyses, by sorting cells from different observed subpopulations. Subsequently, these cells are analyzed by other methods, thus linking different types of information and analytical methods to enhance the understanding of cell behavior.

Cell viability is probably the most widely used parameter in this respect. A comprehensive study of viability assessment by flow cytometry and cell sorting has been described by Nebe-von Caron et al. [7]. By triple fluorochrome staining using propidium iodide, ethidium bromide and bis-oxonol, it is possible to discriminate between undamaged, damaged (membrane depolarized) and dead cells, which was verified by sorting and plating of the different subpopulations. Similar approaches have been followed for lactic acid bacteria [8,9], further underlying the validity of fluorescent viability staining. Comas-Riu and Vives-Rego extended this concept for *Paenibacillus polymyxa *by including the forward scatter signal into the assessment, thus discriminating between live and dead vegetative cells as well as viable and non-viable endospores [10].

Using a similar approach as described above for bacteria, the utility of flow cytometric viability assessment was verified for baker's yeast by sorting and plating [11]. Müller and Lösche analyzed populations of brewing yeast for the content of DNA, neutral lipids and hydroxysterol by flow cytometry, verifying the data with cell sorting and image analysis [12]. Petit et al. described the use of cell sorting (combined with flow cytometry and confocal microscopy) for the study of respiratory dysfunction in yeast [13]. During the last years the distribution of cellular properties, as observed by flow cytometry, and the sorting of specific subpopulations have attracted additional attention for transcriptomic studies. Many biological samples are cell mixtures, and it was shown that sorting the different cell types of model cell mixtures and of cord blood prior to microarray analysis revealed gene expression patterns that were otherwise hidden [14].

## Biotechnological applications of cell sorting

As outlined above, cell sorting is an extremely powerful technique for screening of very large populations of single cells. Therefore it is quite obvious to apply cell sorting to screen for rare events. The potential applications are widespread and very versatile, being confined mainly by the technical potentials of the sorting method. Biotechnological applications can be grouped into either the screening for specific features of biomolecules, mainly proteins, or the screening for cells with specific superior features.

### Protein engineering

**Figure 2:**
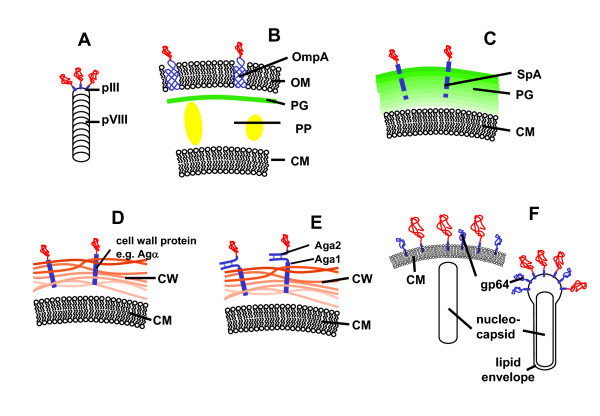
**Pro- and eukaryotic surface display systems**. A: phage display, e.g. phage M13. pIII: minor capsid protein, pVIII: major capsid protein [19]. B: gram negative bacteria, e.g. *E. coli*. Anchor protein: OmpA, CM: cytoplasma membrane, OM: outer membrane, PP: periplasma, PG: peptidoglycan [20]. C: gram positive bacteria, e.g. *Staphylococcus *sp. Anchor protein: SpA: *S. aureus *protein A [21]. D: yeast, direct display, e.g. *S. cerevisiae*. Anchor protein: Agα: agglutinin α. CW: cell wall [22]. E: yeast, indirect display. Aga: agglutinin a: dimeric protein, Aga1 and Aga2, connected by disulfide bonds. Aga1 acts as surface anchor, while the target protein is fused to Aga2 [23]. F: baculovirus (right) and insect cells (left). Anchor protein: major coat protein gp64, which is present on virions and infected insect cells [24].

The optimization of protein structures to improve specific features like enzymatic activity, specificity, binding to ligands, affinity, or stability is an important task for the development of biotechnological products and processes. While rational design of proteins is a challenging task [15], screening of random libraries has been proven to be a valuable alternative in numerous cases (for reviews see [16,17]). A classical random screening approach is phage display of antibody libraries (first described in [18]). Phage display can be regarded as the first example of surface display techniques, which share the common principle that a protein which is encoded in the genome of a host cell (or a virus) is displayed as a fusion protein on the outer surface of the same cell. Consequently, the genetic information is always linked to the respective protein variant in the same cell. Numerous applications of phage and cell surface display have been published. As a comprehensive overview, typical examples of pro- and eukaryotic surface display systems are illustrated in Fig. 2. As a general rule, the target (poly)peptide is fused to a native surface bound protein, be it on a virus capsid, the cell membrane or the cell wall.

#### Screening for binding

Two main features of binding molecules are targets for optimization: specificity and affinity. Phage display (for a comprehensive review see [19]) has proven to be a most valuable tool for the screening for proteins binding specifically to different classes of ligands, and usually employs "panning" as the screening step (reviewed in [25]). The idea of panning is the gold-miners principle of cleaning a sample in a pan (represented by a test tube or a microplate well) from all undesired debris. While panning of phage libraries is a robust and easy to handle method, there exist some handicaps concerning the introduction of diversity, and the screening of kinetics, which can be overcome by employing cell surface expression and FACS (reviewed in [26]). Surface display on bacterial cells [27] and yeast cells [28] has been employed successfully in cell sorting for improved affinity of single chain Fv antibody fragments out of mutant libraries. As an extreme, a dissociation constant K_d _≈ 50 fM was achieved after four rounds of mutagenesis with error prone PCR and FACS screening of 10^5^–10^6 ^cells each [29].

Apart from selecting antibody mutants, surface display and FACS have also been applied for the screening of peptide libraries for ligand binding peptides. *Escherichia coli *surface display was employed in a combined MACS and FACS procedure to isolate peptides that bind with high affinity to different protein ligands [20]. Alternatively, Wernerus and Stahl [21] described the development of a stable *Staphylococcus carnosus *surface display system, its usability for FACS screening, and its application for the screening of ligand binding peptides based on *Staphylococcus aureus *protein A domains, so-called affibody ligands [30].

Viewing beyond the microbial world, baculovirus was exploited as a carrier for surface displayed proteins [31,32]. Peptides or even complex proteins can be displayed either on the virion [31] or on the infected insect cell [33]. Consequently, the selection of displayed peptide sequences was achieved by FACS screening of the Sf9 cells infected with a baculovirus surface display library [34].

#### Screening for enzymatic activity

Screening for ligand binding is quite straight forward in a cell sorting frame, as binding of the fluorescent probe to the cell is an intrinsic feature of the desired setup. On the contrary, a system for the screening of novel or improved enzymatic activities has to be much more elaborate. The major problem to solve is to link the signal (a product of the desired reaction) to the cell that produced an enzyme catalyzing this reaction. While it is rather straightforward to link an enzyme (or a mutated library) to the expressing cell via one of the above described cell surface display systems, the linkage of enzymatic reaction products is more limited and needs consideration in every case. Olsen et al. have employed the negative charge of the cell surface to bind a protease substrate containing a positively charged moiety. The generation of a fluorescence signal upon proteolytic cleavage was enabled by a fluorescence resonance energy transfer (FRET) quenching pair of dyes that was separated upon cleavage, leaving only the fluorescent partner containing25 the positive charges on the cell surface. Using the surface bound serine protease OmpT as a model, a 5000 fold enrichment of *E. coli *expressing active OmpT out of cells expressing an inactive variant could be achieved, as well as the isolation of OmpT variants with an altered substrate specificity with 60 fold enhanced catalytic activity [35]. Recently, this group has demonstrated the simultaneous screening for activity and selectivity by FACS, using again OmpT as a model [36].

An example of intracellular enzyme evolution with the aid of FACS was described by Santoro et al. [37]. Using a cascade of T7 RNA polymerase containing amber stop codons, and green fluorescent protein (GFP), they screened for mutants of aminoacyl-tRNA synthetase finally leading to the incorporation of unnatural amino acids into proteins. Kawarasaki and coworkers [38] described the screening of intracellular activity of glutathione S-transferase using a fluorogenic product that was contained within the cells.

A more radical approach to screening for optimized enzymes is based on cell free transcription and translation in water-in-oil emulsions (in vitro compartmentalization, IVC) [39], which can be also screened by FACS [40]. For a general overview on high throughput screening for enzyme engineering, the reader is referred to [41].

### Cell engineering

Expanding the field beyond protein engineering, one can envisage an extremely wide array of cell engineering applications of single cell sorting. Despite the opportunities to screen for a multitude of different cellular features, cell sorting has been applied so far only in a rather small number of cases to cell engineering.

One of the basic challenges of strain improvement is the isolation of successful hybridization events, e.g. after cell (or protoplast) fusion or cell mating. As an example, FACS was applied to isolate rare yeast mating hybrids without selective markers. The parents strains were stained with two different fluorescent dyes, and a third, double stained population appeared appr. 16 h after mixing, which contained several mated cells [42].

A conceptual expansion would enable the screening for widely different genetic modifications, like the cloning of a random library to a specific position, or of genes with specific desired functions. The dual-fluorescence reporter system described in [43] refers to a method of library screening without plating, by the use of a plasmid encoding two different fluorescent proteins. Upon insertion of a DNA fragment, expression, and hence fluorescence, of one marker is impaired, so that all cells containing a plasmid with insert can be sorted based on the single fluorescence of the remaining dye. There is only a limited number of examples for FACS based cell engineering in the scientific literature, however, some patents describe high-throughput screening for novel bioactivities [44,45]. These methods are based on homologous or heterologous gene libraries employing either intracellular enzyme substrates or gel microdroplet encapsulation of cells. The gel microdroplet technique, first published by Weaver and coworkers 1988 [46] is also described in section 3.3. for use as a method to sort for high production rates. However, it can also be used for physiological studies, measuring individual growth rates, acid production or other physiological parameters [47-49].

Dunn and Handelsman described a method for promoter trapping by cloning a *Bacillus cereus *genomic library upstream of GFP [50]. However, the authors only tested the efficacy of the sorting protocol with cell mixtures containing a 10^4 ^fold excess of non-fluorescent cells (resulting in 95 % fluorescent cells after 3 sorts), but did not prove the applicability of the method to the initial goal of isolating promoters. In a similar approach strong promoter elements of *Mycobaterium smegmatis *were isolated by FACS sorting of a genomic library cloned upstream of a GFP gene [51].

Some other examples are the optimization of protocols for transfection of recombinant cells as well as the selection of new and strong promoters. A destabilized GFP, which does not accumulate in the cells, was used to characterize the kinetics of new promoters or the identification of sequences that enhance secretion [52]. Frequently used tools for this type of research are reporter genes such as β-galactosidase [53] or GFP [54].

While the examples described above relate to broad method development, there are some specific applications of cell sorting to improve cellular characteristics for technological application. Viscardi and coworkers applied cell sorting for immunoselection of phage-resistant *Streptococcus thermophilus *(to be used in the dairy industry). By incubating the cells first with phage, followed by anti-phage antibodies and a fluorescent secondary antibody, the authors could isolate clones that lost interaction with the phage. While it is widely accepted that such a system of "negative staining", i.e. sorting of rare non-fluorescent cells, is more cumbersome and error-prone, these authors were able to successfully isolate desired clones after a single sort [55].

The improvement of cellular properties like viability or stress tolerance may be directly connected to improved overproduction of a desired biotechnological product. As an obvious example, it was demonstrated that the screening for yeast cells with enhanced resistance to weak organic acids in an acidic environment leads directly to strains with enhanced ability to produce lactic acid. Based on the observation that cells with a higher intracellular pH (pH_i_) have a better tolerance to acidic conditions, a FACS sorting strategy was developed. By screening for cells within the highest range of pH_i_, clones with a higher tolerance to acidic environment and a higher productivity of lactic acid were achieved (M. Valli, D. Mattanovich et al., manuscript in preparation).

Other cellular properties that have been selected by cell sorting concern the behaviour of cells during large scale production as well as the stability of recombinant gene expression in CHO cells. Böhm et al. selected for cells with high expression rates during stationary phase or at high cell densities, so that the resulting clones would be better suited for a high density fermentation system [56]. As the production of therapeutic proteins needs to be performed without the usually toxic substances used as selective markers, they also screened for increased stability of recombinant gene expression under these conditions. With a similar objective, a bicistronic vector expressing GFP and interferon gamma was used to sort for cells expressing GFP after serum deprivation under growth arrested conditions [57]. Schlatter and coworkers used a surface marker to sort for proliferation controlled cells, employing both FACS and MACS [58].

Recently, Borth and coworkers were able to select for recombinant CHO cells with altered glycolytic metabolism: cells selected for low mitochondrial membrane potential were found to have lower uptake rates for energy substrates such as glucose and glutamine. At the same time the production rate of lactate was decreased, while the growth rate of cells increased due to the more efficient energy metabolism. Incidentally, the specific production rate for monoclonal antibody was also increased (G. Brugger, N. Borth et al., manuscript in preparation).

### Overproduction

When sorting is used for the selection of over-producing cells, there are three main objectives: (1) to reduce the work load necessary to find over-producers, (2) to reduce the time required and (3) to find the cells with the highest possible production rates, ideally combined with other advantageous properties (see section 3.2.). Brezinsky et al. have nicely illustrated in their work how cell sorting can effectively resolve all three of these objectives [59]. When sorting for high producing recombinant CHO cells, the obtained clones fell into the following categories: 12% very low, 50% low, 32% average and 8% high producers. Clones obtained by limited dilution cloning were 90% very low, 10% low producers, no average or high producers. The timeline for sorting was 6–12 weeks, compared to at least the double with traditional methods, as it was necessary to amplify the gene copy number to reach comparable production rates. Amplification takes more time, it requires another round of subcloning by limited dilution and an additional load of immunoassays for clone testing. In general, simply by eliminating the non- or low-producing cells, sorting will reduce the number of assays to be performed, because only the interesting cells will get to this stage. To obtain stable high producers, it is necessary to isolate single cells, which is not guaranteed by classical limited dilution, because otherwise the faster growing low producers will outgrow any high producers present [60,61].

**Figure 3:**
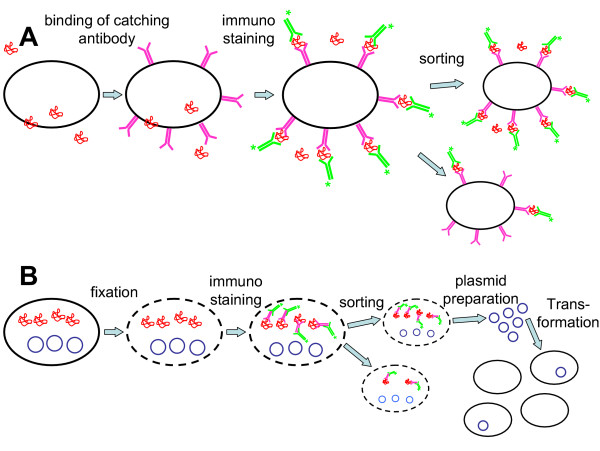
**Immunofluorescence based screening methods for intracellular and secreted proteins**. A: Screening for secreted proteins, developed for mammalian cells. A product specific catching antibody is immobilized on the cell surface, which binds an amount of product proportional to the secretion rate. After staining with a product specific antibody and a secondary antibody, cells with high secretion rate can be sorted and subcloned. B: Screening system for intracellular, plasmid encoded proteins, developed for *E. coli*. Cells are fixed with ethanol and stained with a product specific antibody and a secondary antibody After sorting, plasmids are isolated and retransformed into the host strain.

As many biotechnological products are secreted and thus dissociated from the cells that produced them, three strategies have been described to sort cells directly for high production rates: the first is to catch the product on the surface of the cell that secreted it by applying an artificial matrix on the surface of cells which will bind the product. This affinity matrix approach was establish by Manz and coworkers to study the secretion of immunoglobulin G (IgG) by plasma cells and later on of cytokines by T-cells [62]. It has been used both for the isolation of hybridoma cells [63,64] and for the selection of recombinant CHO cells [65]. An outline of the affinity matrix approach is presented in Fig. 3A. The use of the same approach for yeast cells was less successful for so far unknown reasons [66]. The second strategy was established in 1988 by Weaver et al. and relies on the capture of the secreted product within a microcapsule or gel that surrounds the producer cell [46,67,68]. Finally, Brezinsky et al. developed a strategy that simply "freezes" the product secreted at that moment in the cell membrane by putting the cells on ice. Subsequent staining with a fluorescent antibody against the product allowed to discriminate between cells according to their secretion rate [59].

Before the establishment of these methods, several authors used the correlation found in some hybridoma cell lines between surface expressed IgG and IgG production rate to sort for high producers. This correlation is a specific feature of some hybridoma cell lines, deriving from B-cell development [69-73], and does not apply for recombinant proteins. Native surface expression must be differentiated from the surface capture or entrapment methods described above. Sorting for surface expression directly after hybridoma fusion will loose many specific monoclonal antibody producing cells, which happen to have no surface expression, while both the microcapsule technique and the affinity matrix method have been used successfully for this purpose [64]. Another strategy is the use of GFP-product fusion genes to sort for cells with high GFP production, which will also have high production rates of the gene of interest. However, this strategy has an inherent problem: the cells need to produce two proteins, of which only one is required. This puts a considerable, unnecessary stress on the cell. Although it was shown that the clones with a 3× higher specific product formation rate could be found and that the expression of both product and GFP is reasonably stable, no data on growth rates of these clones or on performance in bioreactors was given [74]. However, these properties are of equal importance for an industrial production cell line as high productivity.

Even though cell fixation to access intracellular product seems to be contradictory, as the cells are necessarily killed prior to sorting, immunofluorescence staining of fixed cells was successfully applied for sorting of *E. coli *cells expressing recombinant superoxide dismutase [75]. As strong overexpression of this as well as many other proteins proved to retard cell growth, the rationale was to screen for promoter mutants out of a mutation library which guide less overexpression, thus enabling continued growth. As the mutation library was localized on a plasmid, it was possible to isolate the plasmids out of fixed sorted cells, retransform them into *E. coli*, thus rescuing the desired promoter variant. Interestingly, most of the sorted promoter variants had multiple point mutations, which essentially cannot be screened by low throughput techniques. Figure 3B outlines the principle of plasmid screening with fixed *E. coli *cells.

While it seems obvious to apply cell sorting for the selection of overproducing clones, the number of published examples dealing with non-protein products is interestingly quite limited. One example is the screening of high producers of polyhydroxyalkanoates (PHAs). Vidal-Mas et al. have described a flow cytometry protocol to measure PHA content of *Pseudomonas aeruginosa *after Nile red and SYTO-13 staining, indicating the utility of this method for cell sorting [76]. Alternatively, Vijayasankaran et al. used heterologous co-expression of GFP in *Pichia pastoris *for the flow cytometry screening of clones with increased overexpression of three PHA biosynthesis genes, thus isolating strains that accumulated appr. 7 % PHA of the cell dry weight instead of 3 % for unsorted strains [77]. As GFP was expressed with an inducible and the PHA biosynthesis genes with a constitutive promoter, the above described problem of an extra load of GFP synthesis during production was avoided. In this setup, GFP was induced only for sorting, while for PHA production the gene was silent. While the same group has developed sensitive staining of PHA by BODIPY 493/503 [78], this probe was apparently not used for sorting.

Several studies have been published on the sorting of plant protoplasts, e.g. for alkaloid production [e.g. [79]]. For more details the reader is referred to references in [80]. In some cases quite unspecific staining has been successfully used for sorting of overproducers. One example is gramicidin S production by *Bacillus brevis*, based on the observation that gramicidin S overproducing cells gave higher fluorescence signals after fluorescein-isothiocyanate (FITC) staining as compared to low or non producers. By sorting for high FITC fluorescence these authors were able to isolate a strain with appr. twofold more gramicidin S production [81]. It should be noted that the potentials of sorting based on autofluorescence or unspecific staining are extensive, hence it is not the intention of this review to list every single application available in the literature.

## Conclusion

While cell sorting is still mainly applied for clinical purposes, there is an increasing interest in biotechnology to utilize its potential for library screening or strain development. The aim of this review was to structure the fields of cell sorting applications in biotechnology, and to highlight many of the examples published over the last years. It turns out that the major fields of use are protein engineering and screening for protein overproduction, both with microbial and animal cells. However, the potential, especially for non-protein products, is still by far not fully utilized.

To design a novel sorting strategy for the improvement of a product or a production strain, the development of a suitable analytical method describing the desired properties adequately is most critical. The full potential of flow cytometry methods, based on autofluorescence, physiological and metabolite specific probes, immunofluorescence, etc. can be utilized for this purpose. After identifying cell populations with superior properties, these can be sorted by FACS, thus directly employing the flow cytometry protocol on a preparative scale. The multiparameter analysis option of flow cytometry is an especially appealing feature, as it enables the simultaneous screening for overproduction and desirable cellular properties like robustness under production conditions. The general screening rule "you get what you screen for" is especially true for cell sorting, which emphasizes the importance of development and design of analytical methods.

By closing the loop back to molecular physiological characterization of sorted strains and to strain development, one can elegantly perform "inverse metabolic engineering" [82]. Gaining understanding of the genetic background of sorted phenotypes enables novel rational approaches for cell and metabolic engineering which may further improve the performance of production strains.

Finally, one may envisage a further refinement of systems biology. At present, most physiological data fed into models were obtained as average values of cell populations. As it becomes more and more obvious that clonal cultures also evolve significant heterogeneities, one can postulate a significant role of flow cytometry and cell sorting in the quantitative description of (multi)cellular systems.

## Competing interests

The author(s) declare that they have no competing interests.

## Authors' contributions

Both authors contributed equally to this manuscript.
